# Integrative analysis reveals PRKCB and SRD5A2 as potential immune-associated biomarkers in prostate cancer targeted by traditional Chinese medicine

**DOI:** 10.3389/fonc.2026.1772573

**Published:** 2026-03-12

**Authors:** Qi Chen, Liang Dong, Wei Xue

**Affiliations:** Department of Urology, Renji Hospital, Shanghai Jiao Tong University School of Medicine, Shanghai, China

**Keywords:** biomarker, immunotherapy, network pharmacology, prostate cancer, traditional Chinese medicine

## Abstract

**Background:**

Prostate cancer (PCa) is a major malignant tumor that significantly threatens male health, in which various immune pathways are critically involved in its pathogenesis. This study aims to elucidate the immunomodulatory mechanisms of active constituents from 11 traditional Chinese medicines in PCa.

**Methods:**

By eliminating batch effects between the GSE69223 and GSE246282 datasets, we integrated weighted gene co-expression network analysis (WGCNA), least absolute shrinkage and selection operator (LASSO) regression, protein–protein interaction (PPI) network analysis, and the CIBERSORT algorithm to identify the active components of traditional Chinese medicines and potential hub immunological biomarkers associated with immune cells. Functional enrichment analyses were conducted using the Kyoto Encyclopedia of Genes and Genomes (KEGG), Gene Ontology (GO), and Gene Set Variation Analysis (GSVA). Molecular docking was employed to evaluate the interactions between active compounds and potential immunotherapeutic targets. In addition, immunohistochemistry was conducted to validate the protein expression of key targets in clinical prostate cancer tissues.

**Results:**

Among the 2,610 differentially expressed genes (DEGs) identified, PRKCB and SRD5A2 emerged as key hub genes. Notably, both genes exhibited significant correlations with immune cell infiltration and showed strong binding affinities to the active compounds kaempferol, isorhamnetin, and rhamnazin. Furthermore, functional enrichment analyses revealed the intricate involvement of the DEGs, hub genes, and candidate biomarkers in pathways closely related to immunity and prostate cancer. Finally, the expression levels of PRKCB and SRD5A2 were validated in prostate cancer and adjacent normal tissues using clinical samples.

**Conclusion:**

PRKCB and SRD5A2 were identified as potential immunological biomarkers and immunotherapeutic targets associated with immune cell infiltration in PCa, both of which were significantly downregulated in PCa tissues. Moreover, kaempferol, isorhamnetin, and rhamnazin exhibited potential immunomodulatory effects in PCa by regulating the expression of PRKCB and SRD5A2.

## 1 Introduction

Prostate cancer (PCa), the second most common malignancy in men worldwide, continues to exhibit rising incidence and mortality rates, posing a significant threat to male health (1–3). Although androgen deprivation therapy (ADT) and novel targeted agents offer therapeutic benefits in the early stages, many patients ultimately progress to metastatic castration-resistant prostate cancer (mCRPC), where treatment is hindered by drug resistance and high recurrence rates (4, 5). In recent years, immunotherapy has emerged as a promising approach for PCa management (6). Advances in tumor immunology have revealed that the onset and progression of PCa are closely linked to immune microenvironment dysregulation, including elevated infiltration of regulatory T cells (Tregs), inadequate infiltration of cytotoxic immune cells, and aberrant activation of immune checkpoint pathways such as PD-L1/PD-1. These alterations contribute to tumor progression and therapeutic resistance (7, 8). As a result, immunomodulatory strategies targeting the tumor immune microenvironment have become a key focus in PCa research.

Traditional Chinese medicine (TCM) presents unique advantages in cancer therapy through its multi-target and multi-pathway synergistic actions (9, 10). The active constituents of TCM have demonstrated antitumor effects, partly by modulating the tumor immune microenvironment (11). Several herbal medicines have shown promising therapeutic potential in the treatment of PCa, including extracts from *Astragalus membranaceus* (AMB), *Tripterygium wilfordii* Hook. f. (TWHF), *Lithospermum erythrorhizon* Siebold & Zucc. (LESZ), *Artemisia annua* L. (AAL), *Scutellaria barbata* D. Don (SBDD), *Andrographis paniculata* (AP), *Fructus Evodiae* (FE), *Gynostemma* Blume (GB), *Curcuma longa* L. (CLL), *Salvia miltiorrhiza* Bunge (SMB), and *Panax notoginseng* (Burk.) F.H. Chen (PFHC) (12–23). Although these herbal medicines have been reported to exhibit anti-PCa activity, their underlying immunomodulatory mechanisms remain poorly understood.

Identifying and elucidating the active components and mechanisms of action of TCM formulas remain major scientific challenges, yet are essential for informing clinical practice and optimizing therapeutic strategies. In recent years, network pharmacology has emerged as an innovative approach, offering new insights and analytical tools to uncover the complex mechanisms of TCM (24, 25). By constructing and analyzing network topologies, this method enables a systematic exploration of the interactions among herbal compounds, target genes, and disease pathways, thereby providing a scientific foundation for the precise clinical application of TCM (26). Furthermore, the integration of bioinformatics has significantly advanced TCM research. Through the incorporation of multi-omics data and computational modeling, researchers can predict and decode the intricate molecular interactions between TCM and disease processes, identify key bioactive compounds, and generate robust evidence to support the therapeutic efficacy of TCM interventions (27).

This study utilized an integrative bioinformatics, network pharmacology, and molecular docking approach to elucidate the immunomodulatory mechanisms of active compounds—notably kaempferol, isorhamnetin, and rhamnazin—from 11 TCM, targeting key PCa biomarkers and immunotherapeutic regulators PRKCB and SRD5A2. By addressing a critical technical bottleneck in the mechanistic interpretation of multi-component TCM formulas, this work provides a visualized network framework to elucidate how TCM modulates the immune microenvironment of PCa. Moreover, it establishes a methodological foundation for a quality control system centered on the “active compound–immune pathway–hub gene” axis. Collectively, these findings advance the field of anti-tumor TCM research from empirical practices toward a more precise and scientifically driven paradigm.

## 2 Materials and methods

### 2.1 Data acquisition and differentially expressed genes analysis

PCa datasets were retrieved from the NCBI GEO database ((https://www.ncbi.nlm.nih.gov/geo) (28), and after filtering for human tissue samples, GSE69223 and GSE246282 were selected for analysis. Data integration and batch effect correction were performed using R software (version 4.4.1). Differential expression analysis between groups was conducted using the limma package to identify DEGs (29).

### 2.2 Functional enrichment analysis and construction of weighted gene co-expression network analysis network

Gene enrichment analyses including Kyoto Encyclopedia of Genes and Genomes (KEGG) (30) and Gene Ontology (GO) were performed using the clusterProfiler package in R software (version 4.4.1). Genes with a p-value < 0.05 and fold change (FC) ≥ 1.5 were included for analysis. Gene Set Variation Analysis (GSVA) was conducted using the GSVA package. WGCNA was performed based on the batch-corrected merged expression matrix of GSE69223 and GSE246282 using the WGCNA package, with a soft-thresholding power β set to 6. Subsequently, key modules and their corresponding hub genes were identified.

### 2.3 Screening of active compounds and targets from 11 TCMs

Active compounds of AMB, TWHF, LESZ, AAL, SBDD, AP, FE, GB, CLL, SMB, and PFHC were obtained from the TCMSP (https://www.91tcmsp.com/#/home) and ETCM (http://www.tcmip.cn/ETCM2/front/#/) databases (31, 32). Oral bioavailability (OB) ≥ 30% and drug-likeness (DL) ≥ 0.18 were used as screening criteria to extract pharmacokinetic data. The main active ingredients and their corresponding target proteins for the 11 herbs were identified (33, 34). Simplified molecular-input line-entry system (SMILES) structures of the active compounds were retrieved from PubChem (https://pubchem.ncbi.nlm.nih.gov/) and analyzed via the SwissADME platform (https://www.swissadme.ch/) (35) to assess gastrointestinal (GI) absorption and drug-likeness. Potential targets were predicted using SwissTargetPrediction (https://www.swisstargetprediction.ch/) (36).

### 2.4 Prediction of PCa-associated targets

PCa-related targets were retrieved by searching the keywords “Prostate Cancer” in the GeneCards (https://www.genecards.org/) (37) and OMIM (https://omim.org/) databases (38). After merging the results from both databases and removing duplicates, a comprehensive set of PCa-associated targets was constructed for subsequent analyses.

### 2.5 Prediction of potential PCa biomarkers

Key genes were identified as potential biomarkers by intersecting DEGs, PCa-associated targets, hub genes, and targets of active compounds. Lasso regression analysis (39, 40) was performed using the glmnet package in R (version 4.4.1), with the lambda value set to 0.02864. Receiver operating characteristic (ROC) curve analysis was conducted using the pROC package, and the area under the curve (AUC) was calculated to evaluate diagnostic performance.

### 2.6 Immune infiltration analysis in PCa

Immune cell infiltration in PCa and normal prostate tissues was estimated using the CIBERSORT algorithm in R (version 4.4.1). Boxplots were used to visualize the distribution of immune cells between PCa and control (CON) groups. To assess differences in immune cell proportions between groups, independent samples t-tests were conducted, with a significance threshold set at α = 0.05.

### 2.7 Construction of the “active compound–immune pathway–hub genes” network and molecular docking

A total of 380 common targets (CTs) were identified by intersecting PCa-related targets with those of the active compounds. Protein–protein interaction (PPI) data for the CTs set were retrieved from the STRING database (https://cn.string-db.org/) (41). By integrating KEGG and GO enrichment pathways, a visual network model was constructed to illustrate the interactions among active compounds, immune pathways, and hub genes. The top three active compounds were identified based on degree centrality within the network. These top-ranked compounds were then subjected to molecular docking with the putative immunotherapeutic targets. Specifically, the 3D structures of the active compounds were obtained from the PubChem database, while the 3D structures of the immune targets were acquired from the UniProt database (https://www.uniprot.org/) (42). Water molecules were removed and hydrogen atoms were added prior to docking, which was performed using AutoDock4 software.

### 2.8 Immunohistochemistry

Postoperative PCa tissue samples were collected from patients at Renji Hospital, Shanghai Jiao Tong University School of Medicine. The donation organization obtains the patient’s consent and signs a written informed consent form. The human tissue collection protocol for this study was approved by the Research Ethics Committee of Renji Hospital (approval number: LY2025-328-B) and all methods were performed in accordance with the ethical standards and according to the Declaration of Helsinki (World Medical Association, 1964), and national and international guidelines. Written informed consent was obtained from all subjects for the collection and use of tissue samples for research purposes. Following fixation, paraffin embedding, and sectioning, the samples underwent immunohistochemical staining. Specifically, tissue sections were deparaffinized, subjected to antigen retrieval, blocked for endogenous peroxidase activity, and incubated with a primary antibody overnight at 4 °C. After three washes with PBS, the sections were incubated with a secondary antibody, followed by color development using diaminobenzidine (DAB) and counterstaining with 10% hematoxylin. The primary antibodies used for IHC were as follows: anti-PRKCB (Absin, Catalog No. abs116899, dilution 1:200) and anti-SRD5A2 (Novus, Catalog No. NBP3-15900, dilution 1:500). Both antibodies are commercially validated for immunohistochemistry. According to the manufacturer’s datasheets, the antibodies were validated by Western blotting, showing a single band at the expected molecular weight. Negative controls confirmed staining specificity.

## 3 Results

### 3.1 Identification and functional enrichment analysis of PCa in DEGs

**Figure 1:**
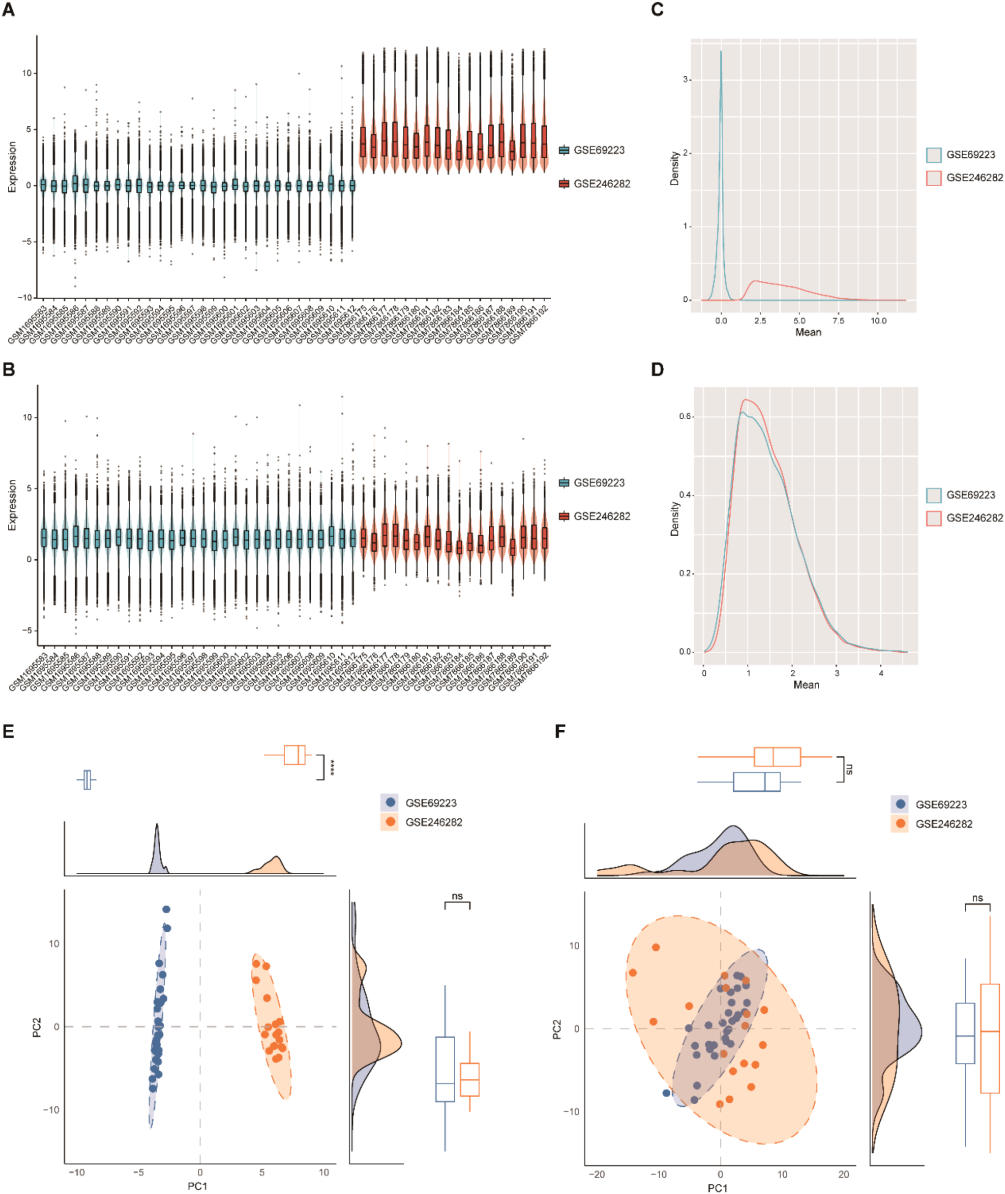
Processing of GEO datasets. **(A)** Gene expression levels of GSE69223 and GSE246282 before batch effect removal. **(B)** Gene expression levels of GSE69223 and GSE246282 after batch effect removal. **(C)** Density distribution prior to batch effect correction. **(D)** Density distribution after batch effect correction. **(E)** PCA before batch effect removal. **(F)** PCA after batch effect removal.

Two prostate cancer gene expression datasets, GSE69223 and GSE246282, were retrieved from the GEO database and selected for integrated analysis. To minimize technical variability, batch effect correction was performed using R (version 4.4.1), and the datasets were subsequently merged (**Figures 1A–F**). Prior to correction, boxplots indicated substantial variation in gene expression distributions between datasets, suggesting the presence of batch effects. After correction, the distributions became uniform across samples (**Figures 1A, B**), as further confirmed by density plots (**Figures 1C, D**). Principal component analysis (PCA) revealed that samples clustered distinctly by dataset before correction but exhibited mixed clustering patterns post-correction, demonstrating successful elimination of batch effects (**Figures 1E, F**).

**Figure 2:**
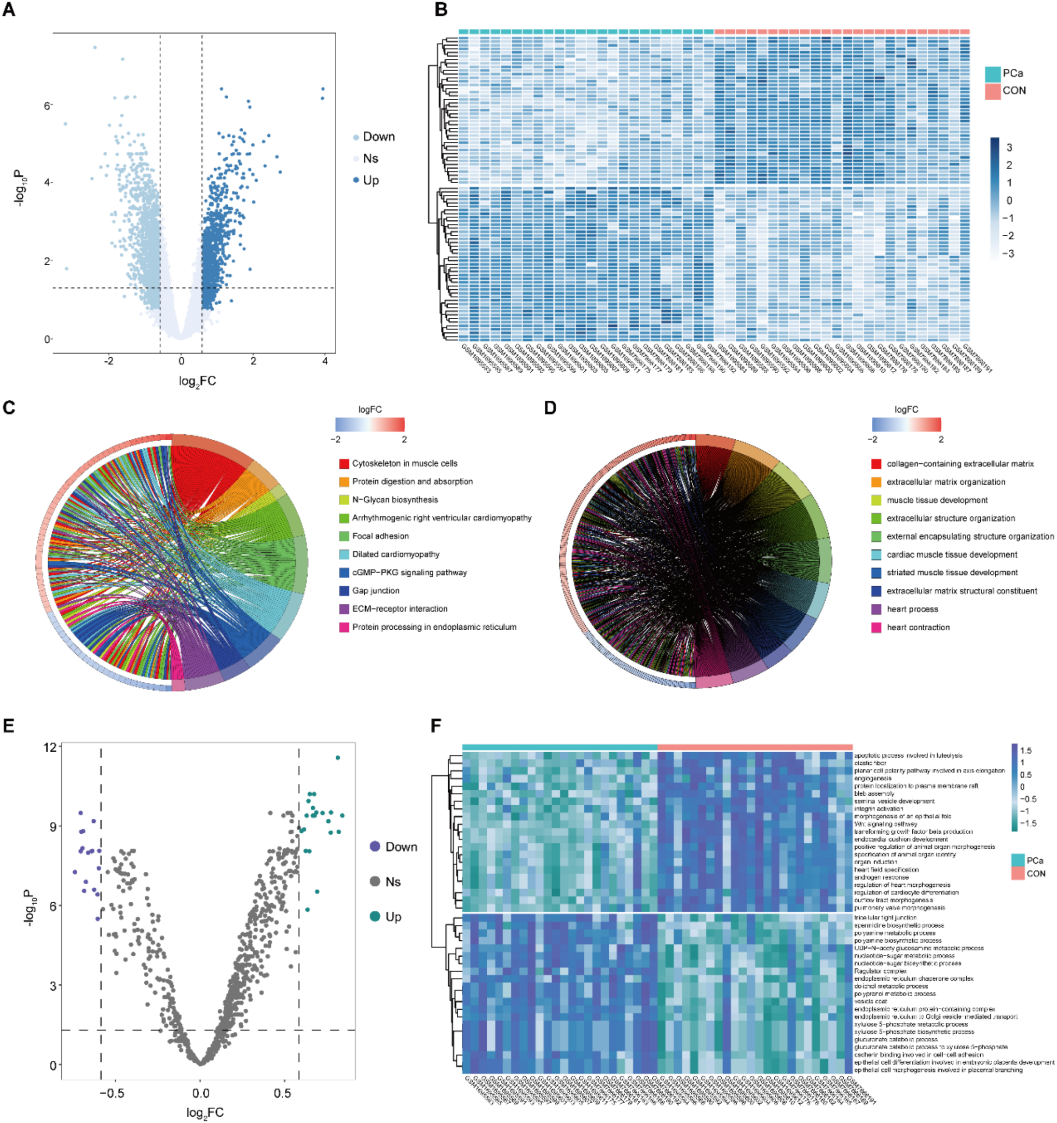
Identification of DEGs and functional enrichment analysis. **(A)** Volcano plot of DEGs. **(B)** Heatmap of DEGs. **(C)** Top 10 enriched KEGG pathways. **(D)** Top 10 enriched GO terms. **(E, F)** GSVA analysis based on GO and KEGG gene sets (30).

Following data integration, samples were categorized into PCa and normal prostate tissue (CON) groups for differential expression analysis. A total of 2,610 DEGs were identified, including 1,059 upregulated and 1,551 downregulated genes in the PCa group (**Figure 2A**). A heatmap was generated to visualize the expression profiles of these DEGs between PCa and CON samples (**Figure 2B**). Functional enrichment analyses were conducted using KEGG and GO frameworks to explore the biological relevance of the identified DEGs (**Figures 2C, D**) (30). The DEGs were significantly enriched in pathways associated with extracellular matrix (ECM) organization and biosynthesis—biological processes closely linked to PCa progression. Notably, the cGMP–PKG signaling pathway, implicated in epithelial-mesenchymal transition (EMT); N-glycan biosynthesis, associated with the development of castration-resistant phenotypes; and the ECM–receptor interaction pathway, known to mediate bone metastasis in PCa, were among the most enriched (43–45).

To further investigate pathway-level activity changes, GSVA was performed, with a particular focus on immune-related signaling pathways (**Figures 2E, F**). Enrichment was observed in pathways involved in TGF-β synthesis, integrin activation, and Wnt signaling—all of which play pivotal roles in immune modulation and tumor progression (46–48). Additionally, pathways associated with tumor invasion, such as angiogenesis and androgen response, were significantly enriched.

### 3.2 Weighted gene co-expression network analysis

**Figure 3:**
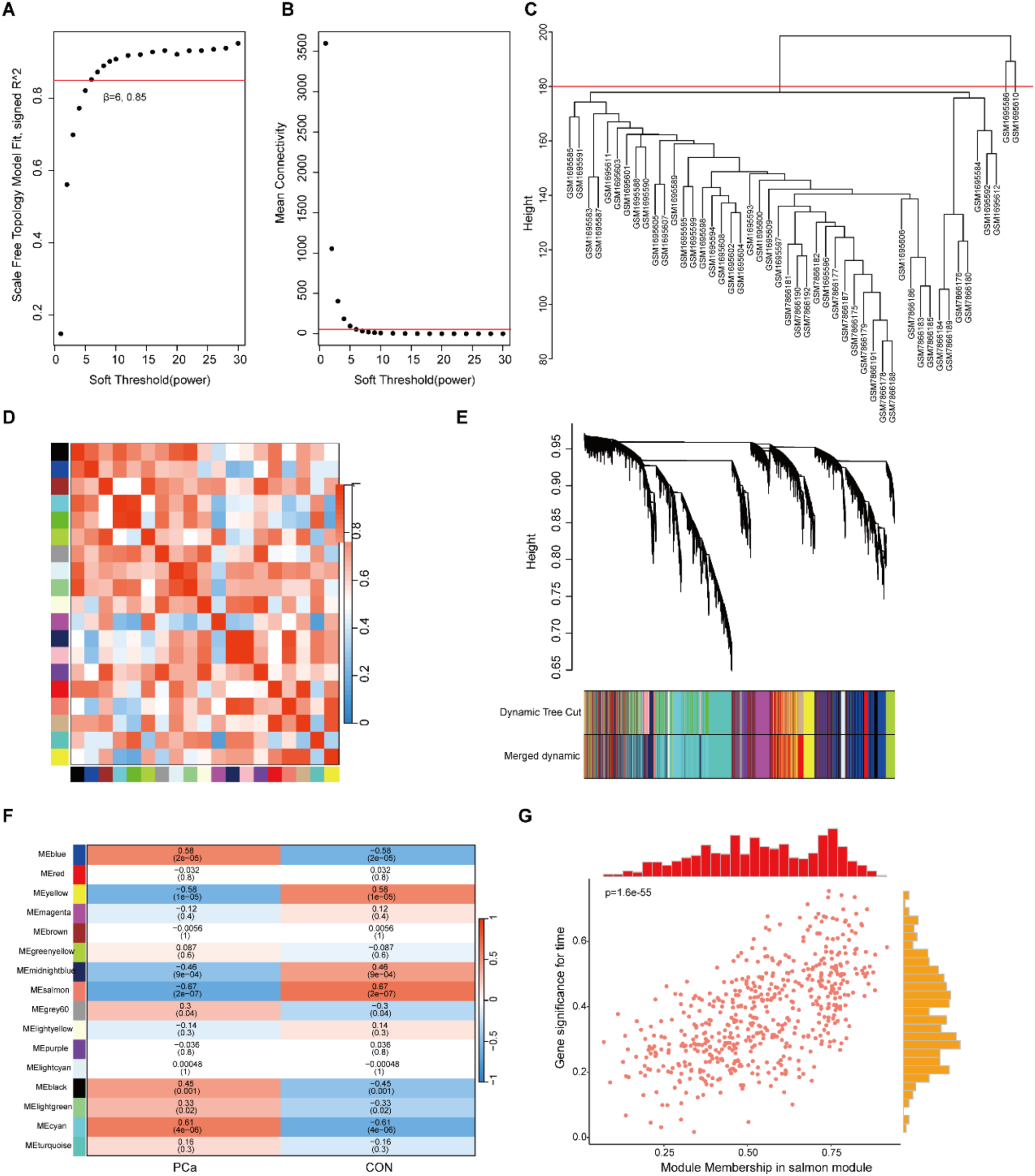
WGCNA analysis of the PCa dataset. **(A, B)** Determination of the soft-thresholding power. **(C)** Sample clustering based on gene expression profiles. **(D)** Heatmap of the topological overlap matrix (TOM). **(E)** Hierarchical clustering dendrogram of genes. **(F)** Correlations between the 16 identified modules and clinical traits. **(G)** Correlation between module membership (MM) and gene significance (GS) in the Salmon module.

To further explore and extract functionally relevant gene modules, we constructed a gene co-expression network using WGCNA (**Figures 3A–G**). Based on the evaluation of scale-free topology and mean connectivity, the optimal soft-thresholding power was set at β = 6 to ensure a scale-free network structure (**Figures 3A, B**). Hierarchical clustering of samples, dynamic tree cutting, and subsequent module detection led to the identification of 16 distinct gene co-expression modules (**Figures 3C–E**). Among these modules, the salmon module demonstrated the strongest correlation with the PCa phenotype (**Figure 3F**). Notably, a significant positive relationship was observed between module membership (MM) and gene significance (GS) within the salmon module, indicating that genes in this module may serve as key regulators in PCa development and could potentially influence treatment outcomes.

### 3.3 Identification of key genes and potential biomarkers

**Figure 4:**
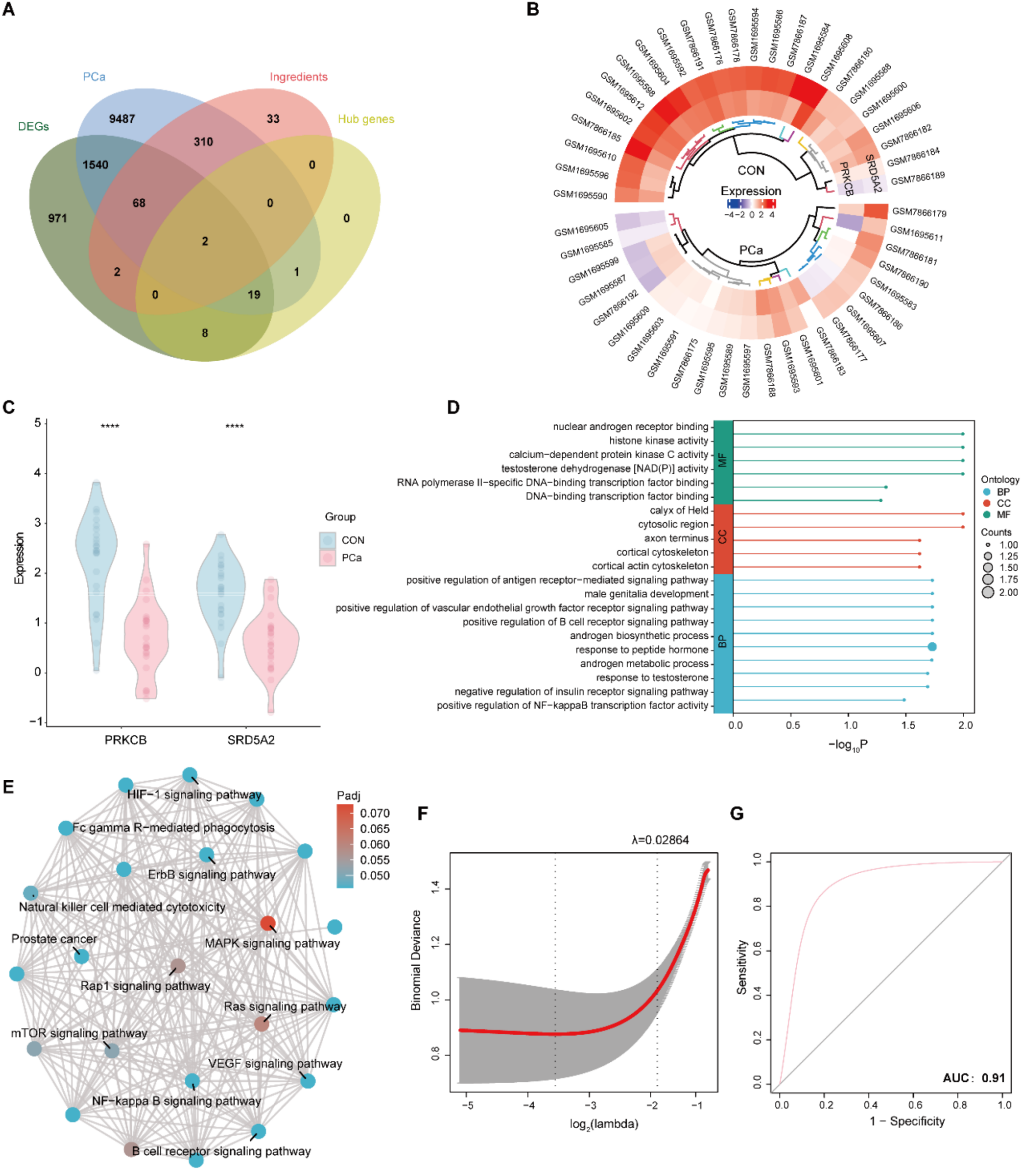
Identification of potential biomarkers. **(A)** Venn diagram of key gene selection. **(B, C)** Heatmap and boxplot showing the expression of key genes across different groups. **(D)** GO enrichment analysis. **(E)** EMAP showing relationships among enriched pathways. **(F)** Construction of a predictive model for potential biomarkers using LASSO analysis. **(G)** ROC curve of the predictive model. ****P < 0.001.

A total of 280 chemical constituents were initially retrieved from publicly available databases for the 11 selected TCMs (**Supplementary Table 1**). Following pharmacokinetic screening using the SwissADME platform—applying oral bioavailability (OB ≥ 30%) and drug-likeness (DL ≥ 0.18) as criteria—170 bioactive compounds were retained for further investigation (**Supplementary Table 2**). Target prediction of these compounds was then performed using SwissTargetPrediction, resulting in the identification of 415 putative protein targets (**Supplementary Table 3**). In parallel, 11,427 PCa-related genes were compiled from the GeneCards and OMIM databases (**Supplementary Table 3**). By intersecting the predicted compound targets, PCa-related genes, DEGs, and hub genes identified via WGCNA, two key genes—SRD5A2 and PRKCB—emerged as potential key genes (**Figure 4A**). Heatmap and boxplot analyses demonstrated significantly decreased expression levels of both genes in PCa tissues compared to normal prostate controls (CON) (**Figures 4B, C**), indicating their possible involvement in disease suppression.

GO enrichment analysis revealed that these key genes were primarily involved in androgen-associated and immune-related signaling pathways, including the NF-κB signaling pathway, VEGF receptor signaling pathway, antigen receptor-mediated signaling pathway, and androgen biosynthetic processes (**Figure 4D**). An enrichment map (EMAP) was constructed to visualize the functional relationships among these enriched pathways (**Figure 4E**). To evaluate the diagnostic utility of the identified genes, a LASSO regression analysis was conducted. The resulting optimal model was defined as: RiskScore = -0.1966 × PRKCB - 0.1797 × SRD5A2. Receiver Operating Characteristic (ROC) curve analysis demonstrated favorable predictive accuracy of the model, supporting its potential clinical value in PCa diagnosis (**Figures 4F, G**).

### 3.4 Immune infiltration analysis in PCa

**Figure 5:**
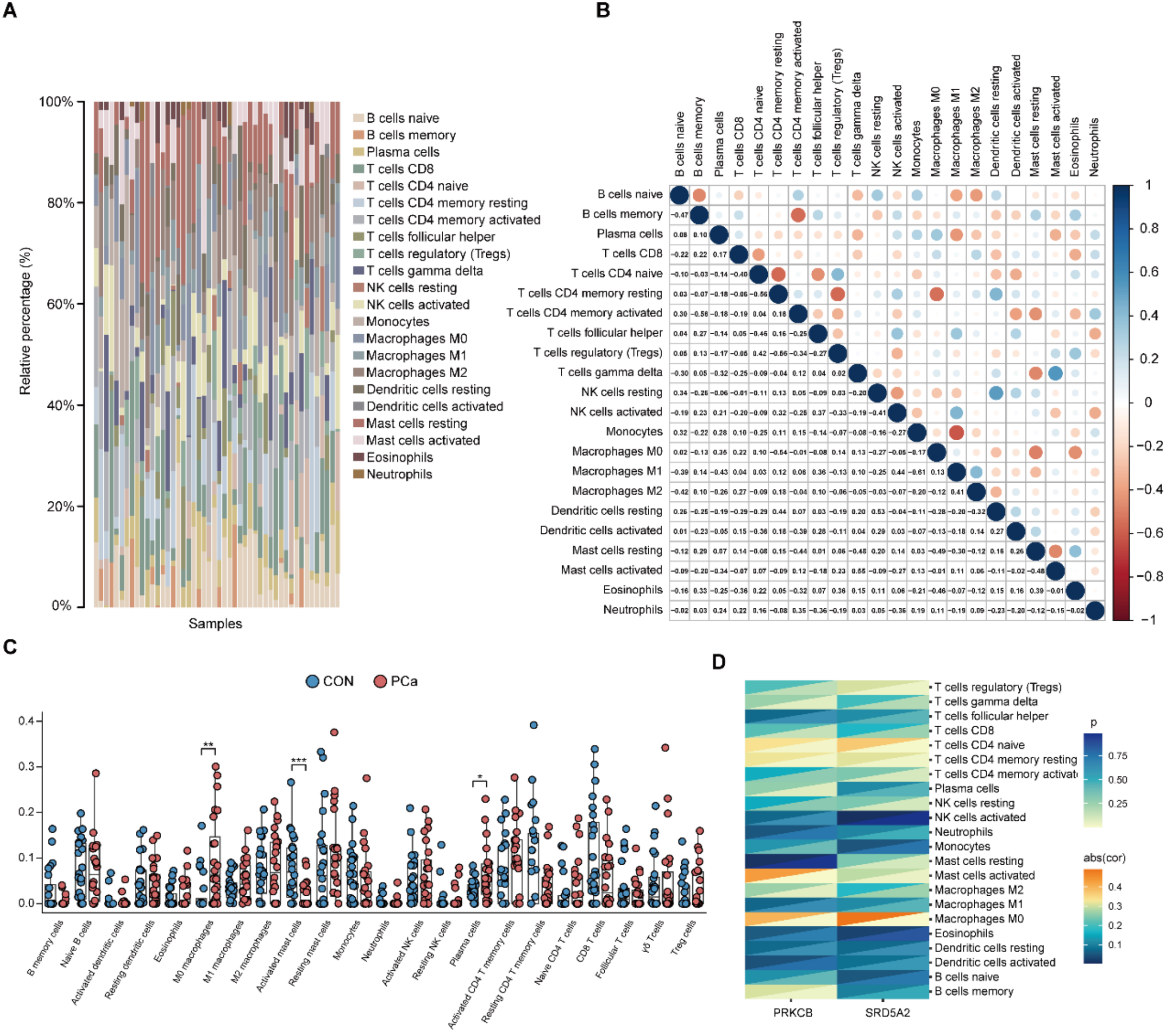
Immune infiltration characteristics in PCa. **(A)** Stacked bar plot showing the proportions of immune cells in PCa and CON samples; the x-axis represents individual samples. **(B)** Heatmap of correlations among different immune cell types in PCa. **(C)** Boxplots showing differences in immune cell infiltration between PCa and CON samples. **(D)** Heatmap of correlations between immune cell types and key gene expression. Data are mean ± SD. Statistical significance in C was determined using one-way ANOVA. *P < 0.05, **P < 0.01, ***P < 0.005.

To elucidate the landscape of immune cell infiltration in PCa, we employed the CIBERSORT algorithm to estimate the relative proportions of 22 immune cell subtypes in both PCa and CON samples from the integrated dataset. The overall distribution of immune cells was visualized using a stacked bar plot, while intercellular correlations were depicted through a correlation heatmap (**Figures 5A, B**). Subsequent differential analysis revealed significant differences in the infiltration levels of M0 macrophages, activated mast cells, and plasma cells between PCa and CON groups (**Figure 5C**). In addition, although the difference did not reach statistical significance, a marked decrease in CD8^+^ T cell infiltration was observed in PCa tissues compared to CON. Correlation analyses further demonstrated that the expression levels of the identified key genes were significantly associated with the abundance of several immune cell types, with the strongest correlation observed for M0 macrophages, suggesting their possible involvement in modulating the tumor immune microenvironment and influencing disease progression (**Figure 5D**).

### 3.5 In-depth analysis of active compounds through PPI network construction

**Figure 6:**
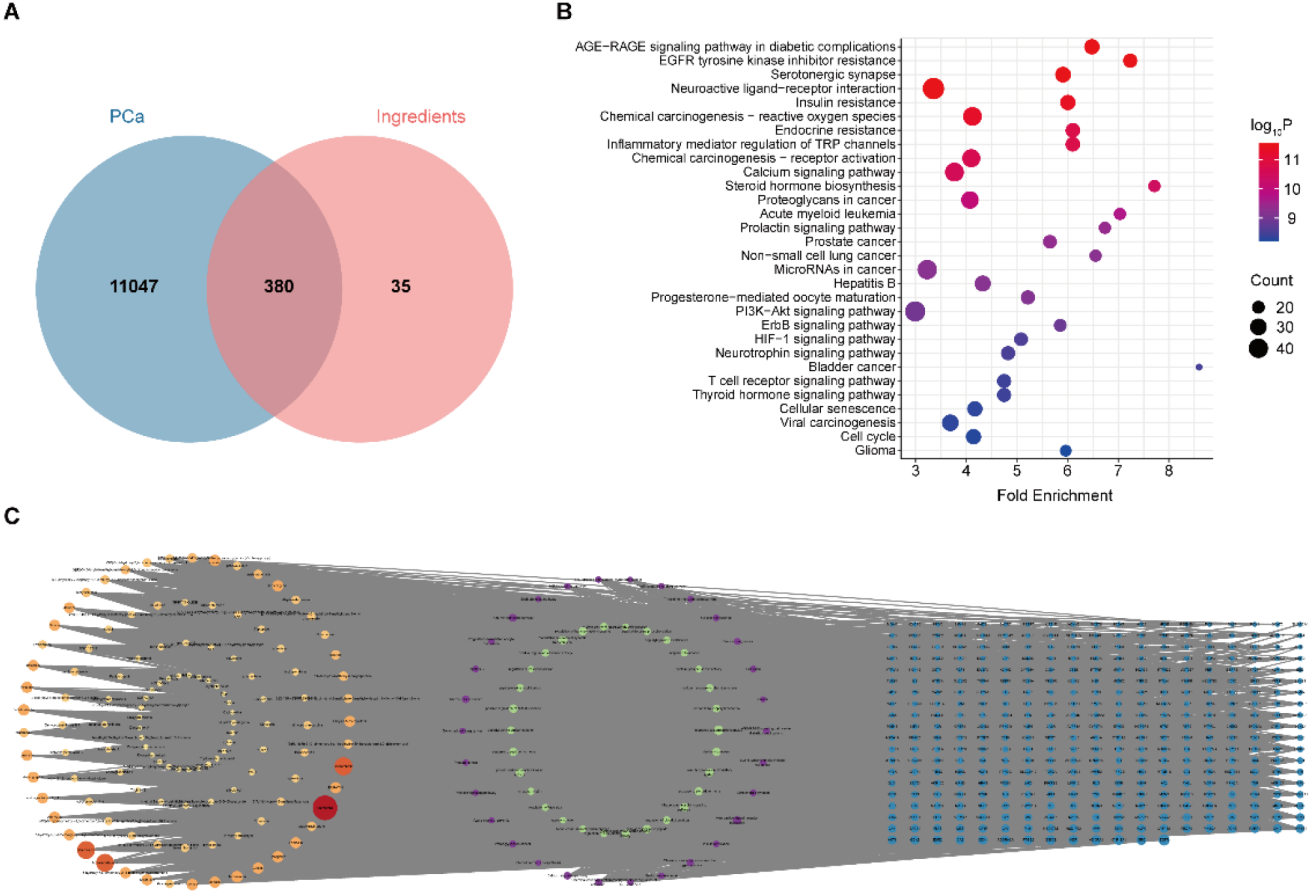
Construction of the active compound–pathway–target network from 11 traditional Chinese medicinal herbs. **(A)** Overlapping targets between PCa-related genes and targets of active compounds. **(B)** Enrichment analysis of the overlapping genes. **(C)** Network diagram illustrating the relationships among active compounds, pathways, and target genes. Blue nodes represent target genes, green and purple nodes represent GO and KEGG enriched pathways, respectively, and orange-red nodes represent active compounds. Edges indicate interactions, and node size is proportional to the degree of interaction.

A total of 380 overlapping genes were identified by intersecting the targets of PCa with predicted compound targets using Venn diagram (**Figure 6A**). Gene enrichment analysis demonstrated that these intersecting genes were predominantly involved in PCa-associated immune-related signaling pathways, including the AGE-RAGE signaling pathway, the EGFR signaling pathway, and the PI3K-Akt signaling pathway (**Figure 6B**). To further elucidate the potential interactions between the 11 bioactive compounds derived from TCM and PCa, a comprehensive “active compound–immune pathway–PCa” network was constructed using Cytoscape (**Figure 6C**). This network offers a systems-level perspective on the complex interplay between bioactive compounds and PCa-related immune mechanisms. Topological analysis of the network, based on degree centrality, identified the five most pivotal compounds: kaempferol, isorhamnetin, rhamnazin, moslosooflavone, and tamarixetin. These compounds are likely to play key roles in modulating immune pathways associated with PCa and warrant further investigation.

### 3.6 Molecular docking and validation

**Figure 7:**
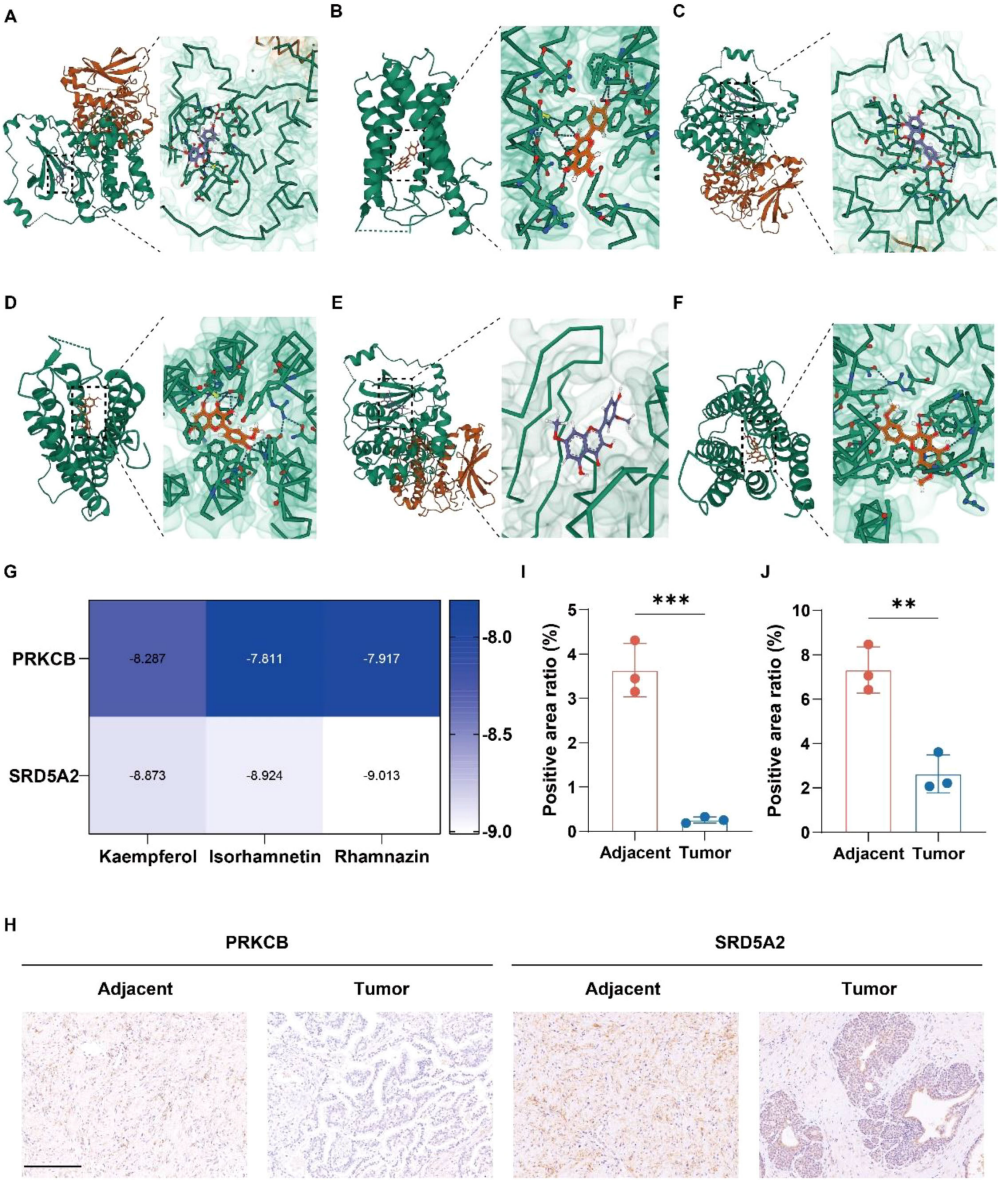
Molecular docking of active compounds from TCM with potential biomarkers and sample validation. **(A)** Molecular docking of kaempferol with PRKCB. **(B)** Molecular docking of kaempferol with SRD5A2. **(C)** Molecular docking of isorhamnetin with PRKCB. **(D)** Molecular docking of isorhamnetin with SRD5A2. **(E)** Molecular docking of rhamnazin with PRKCB. **(F)** rhamnazin with SRD5A2. **(G)** Molecular docking binding energy heatmap. **(H)** Immunohistochemical staining of PRKCB and SRD5A2 in PCa and adjacent non-tumor tissues (n = 3 independent experiments). Scale bar = 200 μm. **(I)** Percentage of PRKCB-positive area in immunohistochemistry (n = 3). **(J)** Percentage of SRD5A2-positive area in immunohistochemistry (n = 3). Data ara mean ± SD. Statistical significance in I and J was determined using two-sided unpaired t-test. **P < 0.01, ***P < 0.005.

To further clarify the immunomodulatory potential of the active compounds in the context of PCa, molecular docking analyses were conducted to evaluate their binding interactions with key immune-related targets (**Figures 7A–F**). The results of molecular docking were visualized by a heatmap, which clearly displayed the binding affinities of the compounds to the target proteins (**Figure 7G**). Based on the network pharmacology findings, the top three active compounds—kaempferol, isorhamnetin, and rhamnazin—were selected for docking with two biomarkers, PRKCB and SRD5A2. The docking simulations revealed favorable binding affinities between each compound and the target proteins, with multiple hydrogen bonds predicted to contribute to the stability of these interactions. Notably, compared with the binding energy of -9.402 kcal/mol of finasteride, a traditional SRD5A2 inhibitor, these small molecules also exhibited extremely high binding stability, suggesting their potential as effective SRD5A2-targeting agents.

To validate the biological relevance of these targets, immunohistochemical (IHC) staining and quantitative analysis were performed on clinical specimens, including human PCa tissues and matched adjacent normal tissues (**Figures 7H–J**). The results demonstrated that PRKCB and SRD5A2 were markedly downregulated in PCa tissues compared to adjacent non-cancerous tissues. These observations suggest that the identified bioactive compounds may exert therapeutic effects by targeting PRKCB and SRD5A2, thereby influencing immune-related signaling pathways involved in PCa progression.

## 4 Discussion

TCM has a rich historical foundation and has demonstrated significant potential in the context of PCa immunotherapy due to its immunomodulatory properties. A growing body of evidence suggests that TCM can enhance antitumor immune responses by modulating the activity of immune cells, regulating immune organs, and influencing the production of cytokines (49, 50). Furthermore, TCM exhibits the capacity to suppress immunosuppressive factors within the tumor microenvironment, thereby contributing to the inhibition of PCa progression (51, 52). Despite its therapeutic promise, the clinical translation of TCM remains hindered by the inherent complexity of herbal formulations and the unpredictable pharmacodynamic interactions among their multiple components. In this regard, network pharmacology offers a powerful approach to systematically identify bioactive compounds and elucidate their underlying molecular mechanisms.

In the present study, we employed a network pharmacology-based strategy to investigate 11 TCM herbs with well-documented antitumor effects and successfully identified three key active compounds—kaempferol, isorhamnetin, and rhamnazin. These findings provide valuable mechanistic insights into how TCM may exert immunotherapeutic effects in the treatment of PCa, and lay the foundation for further exploration of their clinical potential.

An increasing body of evidence suggests that immune evasion and the immunosuppressive state of the tumor microenvironment play critical roles in the progression, metastasis, and therapeutic resistance of PCa (53–55). The tumor microenvironment is a highly dynamic and complex ecosystem composed of tumor cells, immune cells, stromal components, and soluble mediators such as cytokines and chemokines, all of which collectively determine the responsiveness to immunotherapy. In prostate cancer, an immunologically “cold” phenotype is often characterized by limited CD8^+^ T-cell infiltration, increased immunosuppressive populations (e.g., Treg cells and M2-like macrophages), and a cytokine milieu that favors tumor progression (56–60). Therefore, strategies that activate antitumor immune responses and remodel the tumor immune microenvironment hold great promise for the comprehensive treatment of PCa. The regulatory effects of TCM on PCa may, in part, be attributed to its ability to modulate immune responses. In this study, PRKCB and SRD5A2 were identified as potential immune-associated biomarkers and therapeutic targets in PCa. PRKCB is a Ca^2+^/DAG-dependent serine–threonine kinase that functions as a key mediator of BCR/TCR signaling and regulates downstream NF-κB pathways, thereby controlling cytokine production, immune cell activation, and inflammatory responses (61, 62). Dysregulation of PRKCB may impair antitumor immune signaling and contribute to an immunosuppressive tumor microenvironment. Although SRD5A2 is a well-established androgen metabolism enzyme and therapeutic target in PCa, our integrative analysis reveals a previously underexplored association between SRD5A2 expression and immune cell infiltration patterns, suggesting a potential link between androgen metabolism and immune microenvironment modulation (63). Molecular docking analysis further confirmed strong interactions between the active compounds kaempferol, isorhamnetin, and rhamnazin and these two targets. These findings indicate that kaempferol, isorhamnetin, and rhamnazin possess immunomodulatory potential, which may contribute to enhancing the efficacy of PCa immunotherapy.

To explore the underlying mechanisms, GO, KEGG, and GSVA enrichment analyses were conducted to investigate the signaling pathways associated with DEGs and key targets. The results revealed significant enrichment in pathways known to be involved in PCa pathogenesis, including the NF-κB signaling pathway, EGFR signaling pathway, PI3K-AKT signaling pathway, and androgen signaling pathway. PRKCB is a Ca^2+^/DAG-dependent kinase that regulates canonical NF-κB activation and influences macrophage recruitment and polarization, potentially modulating macrophage plasticity and functional status in the tumor microenvironment (62). In addition, PRKCB interacts with PI3K–AKT signaling, which is known to regulate immune cell survival, metabolic reprogramming, and inflammatory responses, thereby shaping the immune landscape in PCa. SRD5A2 catalyzes the conversion of testosterone to dihydrotestosterone, thereby influencing androgen receptor (AR) signaling (48, 64). AR-dependent transcriptional programs can regulate chemokine expression and cytokine secretion, which in turn modulate immune cell infiltration, including macrophages and mast cells (65, 66). Altered androgen metabolism may therefore contribute to changes in immune cell distribution and immune suppressive states in PCa. Therefore, targeting PRKCB and SRD5A2 through immunotherapeutic approaches may modulate immune infiltration within the tumor microenvironment and offer a mechanistic basis for understanding the immunotherapeutic potential of kaempferol, isorhamnetin, and rhamnazin.

However, several limitations should be acknowledged. Our conclusions are based chiefly on integrative bioinformatics analyses and validated by tissue immunohistochemistry, which together establish robust associations but do not prove causation. We did not perform functional perturbation experiments such as gene knockdown/overexpression or *in vivo* studies to demonstrate that TCMs modulate immune responses via PRKCB/SRD5A2; therefore, these genes should be interpreted here as immune-associated biomarkers rather than definitive immunoregulatory drivers. Future work including targeted manipulation of PRKCB and SRD5A2 in PCa models, mechanistic pathway interrogation, and validation in larger clinical cohorts will be required to determine their causal roles in shaping immune infiltration and therapeutic responsiveness.

## 5 Conclusion

In conclusion, this study identified PRKCB and SRD5A2 as potential biomarkers and immunotherapeutic targets for PCa, and further explored the immunomodulatory effects of active compounds—kaempferol, isorhamnetin, and rhamnazin—derived from 11 traditional Chinese medicinal herbs. These findings provide valuable insights into the molecular mechanisms underlying the use of TCM in PCa immunotherapy and lay a solid foundation for the development of novel immunotherapeutic strategies for PCa in the future.

## Data Availability

The datasets presented in this study can be found in online repositories. The names of the repository/repositories and accession number(s) can be found below: https://www.ncbi.nlm.nih.gov/geo/, GSE69223 https://www.ncbi.nlm.nih.gov/geo/, GSE246282.
